# Alternative dosing regimen of exemestane in a randomized presurgical trial: the role of obesity in biomarker modulation

**DOI:** 10.1038/s41523-024-00616-8

**Published:** 2024-01-18

**Authors:** Harriet Johansson, Aliana Guerrieri-Gonzaga, Sara Gandini, Bjørn-Erik Bertelsen, Debora Macis, Davide Serrano, Gunnar Mellgren, Matteo Lazzeroni, Parijatham S. Thomas, Katherine D. Crew, Nagi B. Kumar, Irene Maria Briata, Viviana Galimberti, Giuseppe Viale, Lana A. Vornik, Valentina Aristarco, Tania Buttiron Webber, Stefano Spinaci, Powel H. Brown, Brandy M. Heckman-Stoddard, Eva Szabo, Bernardo Bonanni, Andrea DeCensi

**Affiliations:** 1 IEO, European Institute of Oncology IRCCS, Milan, Italy; 2 Hormone Laboratory, Department of Medical Biochemistry and Pharmacology, Haukeland University Hospital, Bergen, Norway; 3 Department of Clinical Science, University of Bergen, Bergen, Norway; 4 University of Texas, MD Anderson Cancer Center, Houston, TX USA; 5 Columbia University Irving Medical Center, New York, NY USA; 6 Moffitt Cancer Center, University of South Florida, Tampa, FL USA; 7 E.O. Galliera Hospital, Genoa, Italy; 8 Ospedale Villa Scassi ASL3, Genoa, Italy; 9 Division of Cancer Prevention, NCI Bethesda, MD, USA; 10 Wolfson Institute of Population Health, Queen Mary University of London, London, UK

**Keywords:** Cancer prevention, Randomized controlled trials

## Abstract

In a 3-arm presurgical trial, four-six weeks exemestane 25 mg three times/week (TIW) was non-inferior to 25 mg/day (QD) in suppressing circulating estradiol in postmenopausal women with ER-positive breast cancer. Since obesity may decrease exemestane efficacy, we analyzed changes in sex steroids, adipokines, Ki-67, and drug levels in relation to obesity. Postmenopausal women with early-stage ER-positive breast cancer were randomized to either exemestane 25 mg QD (*n* = 57), 25 mg TIW (*n* = 57), or 25 mg/week (QW, *n* = 62) for 4–6 weeks before breast surgery. Serum and tissue pre- and post-treatment biomarkers were stratified by body mass index (BMI)< or ≥30 kg/m^2^. Post-treatment median exemestane and 17-OH exemestane levels were 5–6 times higher in the QD arm compared to the TIW arm. For obese women, TIW maintained comparable reductions to QD in systemic estradiol levels, although the reduction in estrone was less with the TIW regimen. There was less suppression of SHBG with the TIW versus the QD dose schedule in obese women which should result in less systemic bioavailable estrogens. Metabolically, the effect of the TIW regimen was similar to the QD regimen for obese women in terms of leptin suppression and increase in the adiponectin-leptin ratio. Reduction in tissue Ki-67 was less for obese women on the TIW regimen than QD, although changes were similar for non-obese women. Our findings suggest that TIW exemestane should be explored further for primary cancer prevention in both normal weight and obese cohorts.

## Introduction

Breast cancer is the most commonly diagnosed cancer among women, and a moderate increase in incidence rates has occurred since the first decade of the 21^st^ century^1^. Explanations include the obesity epidemics in relation to its strong association with estrogen receptor (ER)-positive cancer among postmenopausal women, underlining the emerging need to increase early detection and cancer prevention uptake. Specifically, preventive therapy, including selective estrogen receptor modulators^2^, and aromatase inhibitors^3,4^ is a valuable option in women at increased risk^5^.

Exemestane is an irreversible steroidal inactivator of the aromatase enzyme that is effective in all settings, from prevention to adjuvant and metastatic breast cancer treatment^6^. By its androstenedione-like structure, exemestane competes with the natural substrates androstenedione and testosterone to ultimately form covalent bonds with the substrate-binding site of the aromatase enzyme, causing permanent inactivation. However, a major issue is the adherence to aromatase inhibitors in the adjuvant setting which is hampered by adverse events that may lead to drop-out and a decrease in treatment efficacy, such as menopausal symptoms, arthralgias, and an increased risk of osteoporosis^7^.

Our experience with low-dose tamoxifen has shown retained efficacy and lower toxicity in preventing recurrence from non-invasive breast cancer^8^. Indeed, getting the dose right and optimizing dose selection strategies in oncology according to specific clinical situations or populations is an important paradigm shift recently endorsed by the U.S. Food and Drug Administration (FDA) and the American Society of Clinical Oncology (ASCO)^9^. However, in obese postmenopausal women, the increased production of estrogen by aromatase in excessive adipose tissue represents an important issue. Some studies have reported suboptimal efficacy with aromatase inhibitors in women with overweight or obesity^10–12^, although evidence is contradictory^13^ and recommendations for tailoring adjuvant endocrine therapy are lacking^14^. Thus, studies investigating the option of personalized drug choice and dose de-escalation should be considered for optimal treatment efficacy, along with reduced adverse events^9^.

In a recent randomized three-arm international presurgical trial, we have shown the non-inferiority of exemestane 25 mg administered three times a week (TIW), compared to exemestane 25 mg/daily (QD), in maintaining estradiol suppression and decreasing the breast cancer tissue proliferation index Ki-67^15^. Specifically, in compliant patients, representing 90% of the overall study population, the TIW schedule was able to retain modulation similar to the standard dose concerning circulating estradiol, estrone, total estrone, Ki-67, and progesterone receptor (PgR) expression, whereas the 25 mg once-a-week schedule (QW), was inferior on most biomarkers. Here we focus on the interaction between body mass index (BMI, non-obese versus obese) and alternative schedule activity of exemestane assessed by measuring drug concentrations, estradiol, estrogen, sex hormone binding globulin (SHBG), androstenedione, testosterone, breast cancer tissue Ki-67, adipokines, and insulin sensitivity markers.

## Results

### Characteristics of the participants

**Fig. 1:**
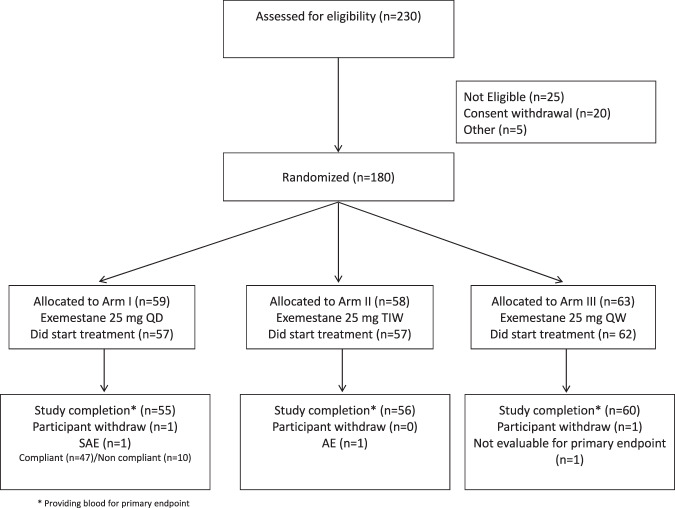
Consort diagram.

**Fig. 2:**
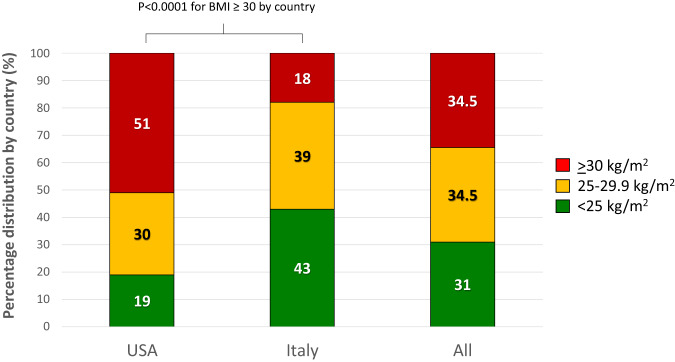
Percentage distribution of body weight categories (kg/m^2^).

The CONSORT diagram is depicted in Fig. 1. Baseline patient characteristics have been published elsewhere^15^. Median age and interquartile range (IQR) at study entry were 66 years (60 to 71) in the QD arm, 63 years (60 to 69) in the TIW arm, and 65 years (61 to 70) in the QW arm. Overall, 34% of women were obese, with frequencies evenly distributed among arms, but a considerable difference was observed between the American and Italian cohorts. Specifically, in the US cohort, the majority of women were obese (51%) and only a small proportion was normal weight (19%), while the opposite distribution was observed for the Italian cohort, where the percentage of obese women was only 18%, and up to 43% were normal weight (*p* < 0.0001, Fig. 2).

### Serum drug and biomarker changes by BMI category and arm

Firstly, we investigated the relationship between drug concentrations and endpoint biomarkers by treatment arm and BMI category (non-obese versus obese) as shown in Table 1. Expectedly, obese women had markedly higher baseline levels of estrogens. Post-treatment median drug and metabolite levels were 5–6 times higher in the QD arm compared to the TIW arm. Also, there was a tendency for obese women to have higher drug levels than non-obese women in the QD arm. Post-treatment serum estradiol levels did not differ between QD and TIW obese women despite the lower levels of exemestane and its 17-OH metabolite. However, post-treatment estrone levels were higher for QD vs TIW (*p* = 0.004). There was no significant difference in post-treatment estradiol or estrone levels in non-obese women between QD and TIW. Both post-treatment estradiol and estrone levels were higher for QW than QD.

**Table 1:** Median and interquartile ranges of post-treatment serum exemestane, 17-OH- exemestane, and baseline and absolute changes in circulating estrogens, SHBG, and tumor Ki-67 by BMI stratification (non-obese and obese).

	Exemestane 25 mg QD		Exemestane 25 mg TIW		Exemestane 25 mg QW		P-value BMI < 30		P-value BMI ≥ 30	
Variable	BMI < 30 (*n* = 34)	BMI ≥ 30 (*n* = 19)	BMI < 30 (*n* = 38)	BMI ≥ 30 (*n* = 16)	BMI < 30 (*n* = 37)	BMI ≥ 30 (*n* = 22)	QD vs TIW	QD vs QW	QD vs TIW	QD vs QW
Exemestane (pmol/L) post-treatment	2928 (1981; 4458)	3355 (2922; 4567)	520 (381; 826)	541 (357; 682)	26 (6.5; 46)	22 (17; 35)	**<0.0001**	**<0.0001**	**<0.0001**	**<0.0001**
17-OH-exemestane (pmol/L) post-treatment	922 (644;1381)	1419 (600; 1865)	229 (119; 345)	228 (154; 406)	10 (4; 38)	4 (4; 22)	**0.0004**	**<0.0001**	**0.0003**	**<0.0001**
Estradiol baseline (pmol/L)	16 (12; 24)	36 (24; 56)	16 (11; 28)	28 (22; 46)	16 (11; 23)	24 (22; 38)	0.946	0.731	0.502	0.141
Estradiol absolute change	−14.6 (−23.2; −11.6)	−35.4 (−53.6; −22.6)	−13.8 (−27.1; −8.5)	−26.3 (−44.7; −21.6)	−9.7 (−16.2; −6.4)	−20.1 (−26.2; −14.9)	0.283	**0.009**	0.081	**<0.0001**
Estrone baseline (pmol/L)	89 (67; 129)	116 (78; 152)	86 (62; 127)	128 (85; 165)	86 (67; 112)	122 (83; 142)	0.906	0.779	0.622	0.957
Estrone absolute change	−87 (−125; −65)	−115 (−150; − 73)	−83 (−118; −61)	−121 (−158; −81)	−61 (−83; −34)	−87 (−113; 62)	0.462	**<0.0001**	**0.004**	**<0.0001**
SHBG baseline (nmol/L)	53.9 (36.3; 65.8)	53.4 (41.8; 60.8)	51.7 (38.3; 66.9)	41.4 (29.3; 55.8)	55.9 (39.4; 75.5)	33.0 (22.8;50.3)	0.081	0.563	0.211	**0.006**
SHBG absolute change	−11.4 (−20.1; −8.2)	−14.5 (−20.7; −9.1)	−9.2 (−14.6; −3.8)	−3.4 (−7.1; −0.04)	−1.5 (−8.0; 1.6)	−2.3 (−5.3; −0.4)	0.085	**<0.0001**	**<0.0001**	0.078
Ki-67 baseline (%)	12.5 (9; 17)	11.5 (7; 17)	12 (7; 21)	15 (8; 18)	11 (5.5; 18)	10.5 (7; 19)	0.860	0.392	0.496	1
Ki-67 absolute change	−6 (−10; −3)	−8 (−13; −3)	−5 (−11; −1)	−4 (−9; −2)	−2 (−6; 0)	−5 (−8; −2)	0.700	0.130	**0.020**	**0.037**

Bold values indicate a statistically significant difference between arms.

In obese women, there was less suppression of SHBG in the TIW arm than the QD arm (−14.5 vs −3.4 median absolute change, *p* < 0.0001), while no difference was observed in non-obese.

Leptin levels decreased differently with exemestane according to the BMI category (Table 2). Leptin levels were decreased with exemestane but not markedly influenced by the dose regimen. The absolute decreases were greater in obese women. Adiponectin was reduced in both obese and non-obese women. The reduction in adiponectin (unfavorable) appeared to be less for the TIW versus the daily dosing regimen for obese and non-obese women. There was little change in the adiponectin/leptin ratio. Insulin and HOMA did not exhibit any statistically significant change among arms regardless of BMI.

**Table 2:** Median and interquartile ranges of baseline and absolute changes in adipokines, insulin, and HOMA index by BMI stratification (non-obese and obese).

	Exemestane 25 mg QD		Exemestane 25 mg TIW		Exemestane 25 mg QW		*P*-value BMI < 30		*P*-value BMI ≥ 30	
Variable	BMI < 30 (*n* = 34)	BMI ≥ 30 (*n* = 19)	BMI < 30 (*n* = 38)	BMI ≥ 30 (*n* = 16)	BMI < 30 (*n* = 37)	BMI ≥ 30 (*n* = 22)	QD vs TIW	QD vs QW	QD vs TIW	QD vs QW
Leptin (L) baseline (ng/mL)	29.2 (18.6; 36.6)	80 (47.6; 104.8)	28.7 (14.4; 44.7)	73.8 (59.0; 82.4)	27.9 (14.2;38.5)	78.6 (63.1; 98.0)	0.715	0.574	0.523	0.978
Leptin absolute change	−5.8 (−10.9; −2.5)	−10.9 (−31.2; −1.1)	0 (−8.3; 2.9)	−12.6 (−20.5; −5.3)	−1.4 (−5; 4.4)	−4.5 (−13.2; 7.6)	**0.015**	**0.047**	0.739	0.066
Adiponectin (A) baseline (µg/mL)	12.1 (10.2;15.0)	8.0 (4.8; 11.8)	8.8 (6.7;11.9)	7.3 (5.2; 11.5)	8.6 (6.5; 12.9)	**0.005**	**0.013**	0.831	0.629	**0.005**
Adiponectin absolute change	−1.36 (−2.32; 0.3)	−0.86 (−1.71; −0.2)	−0.26 (−0.9; 0.76)	−0.06 (−0.54; 0.34)	−0.16 (−1.00; 0.69)	0.01 (−0.40; 0.67)	**0.030**	0.554	**0.049**	**0.006**
A/L ratio absolute change	0.049 (−0.013; 0.187)	0.016 (−0.011; 0.064)	−0.013 (−0.060; 0.058)	0.020 (−0.001; 0.060)	0.003 (−0.093; 0.049)	0.000 (−0.029; 0.034)	**0.045**	0.110	0.749	0.118
Insulin baseline (uU/mL)	7.1 (5.7; 10.6)	8.4 (5.9; 11.4)	7.6 (5.9; 10.9)	11.6 (6.5; 17.8)	8.0 (4.8; 11.0)	7.0 (6.7; 13.1)	0.626	0.874	0.252	0.807
Insulin absolute change	0.2 (−1.3; 3.7)	0.6 (−0.7; 3.3)	0.7 (−1.1; 3.1)	0.6 (−1.3; 3.2)	0.4 (−1.1; 1.9)	−0.2 (−5.8; 3.5)	0.617	0.671	0.459	0.687
HOMA baseline	1.5 (1.3; 2.6)	2.1 (1.5; 3.0)	1.9 (1.4; 2.8)	3.4 (1.6; 6.2)	2.0 (1.2; 2.8)	1.7 (1.4; 3.6)	0.359	0.800	0.239	0.957
HOMA absolute change	0.08 (−0.37; 0.90)	0.40 (−0.47; 1.26)	0.08 (−051; 0.63)	0.50 (−0.76; 1.52)	0.08 (−0.44; 1.00)	0.10 (−0.98; 0.81)	0.732	0.186	0.363	0.796

Bold values indicate a statistically significant difference between arms.

### Tissue biomarker changes

**Fig. 3:**
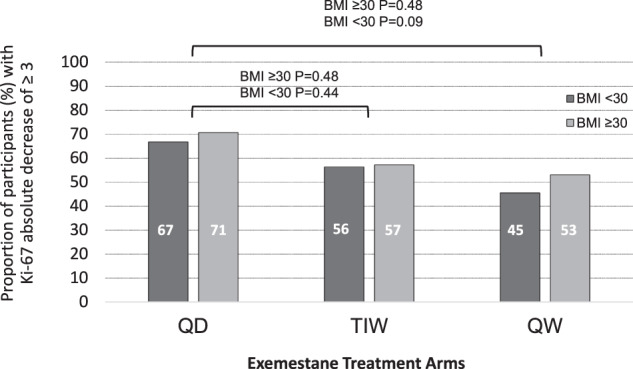
Proportions of participants having an absolute decrease in tumor Ki-67 of at least 3 units by treatment arm at the surgery.

Although the median decrease in Ki-67 change in non-obese participants was similar among the three dosing regimens, the median reduction of 4% in Ki67 in the TIW dose schedule in obese women was less than the 8% median reduction in the QD arm which was marginally significant (*p* = 0.02). Importantly, adopting the short-term threshold of at least −3% in Ki-67 absolute change as a clinically relevant cut-off^16^, the proportions of obese participants that reached a reduction in Ki-67 of −3 or more (QD arm 71%; TIW arm 57%; and QW arm 53%) were even higher than in the non-obese in all arms (QD arm 67%; TIW arm 56%; and QW arm 45%) (Fig. 3). No changes were observed for testosterone or androstenedione among arms (Supplementary Table 1), except for a slight increase in androstenedione in the TIW arm compared to the QD arm in obese participants.

**Fig. 4:**
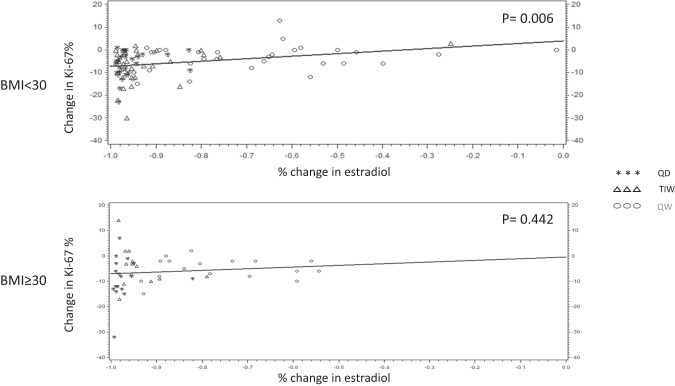
Predicted and observed Ki-67% absolute change by % change of serum estradiol according to BMI stratification (non-obese and obese).

**Fig. 5:**
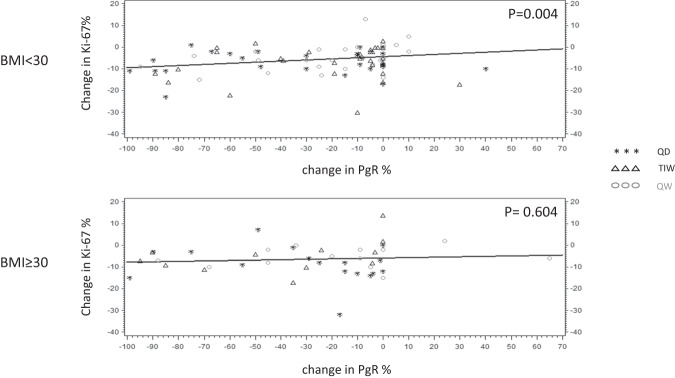
Predicted and observed Ki-67 absolute change by absolute change in PgR % expression according to BMI (non-obese and obese).

In addition, we investigated the association of serum estradiol percentage change (Fig. 4) and PgR breast tissue expression percentage change (Fig. 5) with Ki-67 absolute change according to BMI stratification (non-obese versus obese). We found a highly significant correlation between estradiol change (*p* = 0.006), or PgR change (*p* = 0.004) and Ki-67 change in non-obese women, as opposed to obese women, where neither estradiol change (*p* = 0.422), nor PgR change (*p* = 0.604), correlated with Ki-67 absolute change.

## Discussion

Concerns regarding aromatase inhibitor efficacy in obese women have been raised, although several large adjuvant studies do not support the hypothesis that obese patients have worse outcomes^17,18^ and no specific recommendations have been suggested^13,14^. Given the association of BMI with postmenopausal breast cancer risk, linked to the associated increase in bioavailable estradiol^19^, we aimed to investigate the interaction of BMI with exemestane alternative schedule activity. Despite our findings of five to six times lower serum drug concentrations versus QD, the retained estradiol suppression of TIW suggests that exemestane is extensively distributed into the peripheral tissues. Like other highly lipophilic steroids, exemestane may concentrate^20^ in the adipose tissue^20^, prolonging exemestane activity locally. Additionally, as an irreversible steroidal inactivator of the aromatase enzyme, the drug is expected to maintain its activity even after clearance from circulation^21^. Interestingly, despite markedly lower serum concentrations of exemestane and metabolite compared to the full dose, 25 mg TIW maintained comparable activity on serum estradiol decrease in both non-obese and obese women. Yet, serum estrone and Ki-67 were less effectively suppressed in the TIW arm compared to the QD arm in obese patients (*p* = 0.004 and *p* = 0.02, respectively). Estrone is a precursor to estradiol and may affect the estradiol concentrations at the breast cancer tissue level^22,23^. Indeed, the current analysis is exploratory since the trial was not powered to investigate this subgroup effect and the proportion of obese women (*n* = 57) was lower than the proportion of non-obese. Nonetheless, considering that some aromatase inhibitors may not perform as well in the setting of obesity due to the higher levels of tissue aromatase and estrogen^24^, our results appear relevant. BMI modifies the efficacy of anastrozole^10,11^ but not letrozole^17^, or exemestane^25^, possibly due to a lower potency of anastrozole in suppressing estrogens^24,26^. Thus, letrozole or exemestane may be the preferred aromatase inhibitors in women with obesity. When considering the clinical threshold of at least −3% absolute change in Ki-67 after short-term treatment^16^, the proportion of obese participants that reached this threshold was even higher than in non-obese in all three arms. This finding points to a favorable role of exemestane on the inactivation of aromatase tissue activity even at reduced dose regimens.

A dose-response decrease in SHBG was observed in favor of the TIW dose as opposed to the stronger decrease with the standard dose, mainly in obese women, while the difference between dose schedules did not reach statistical significance in non-obese subjects. SHBG plays a critical role in regulating the bioavailability of estradiol through its high-affinity binding sites^27^. Acting as a buffer and gatekeeper of steroid activity it has been shown to protect against endocrine-responsive breast cancer^28^. The SHBG reduction with the TIW dose schedule was indeed small in obese women, as opposed to the marked decrease with the full dose schedule. In consideration of a less efficacious estrone suppression in this category, SHBG may partially contribute to reducing the bioavailability of estrone in this subgroup. Serum SHBG, mainly of hepatic origin^29^, is inversely associated with BMI^19^ along with several metabolic disorders, including polycystic ovary syndrome (PCOS), and type 2 diabetes, but a causal link by SHBG with these disorders remains to be proven^29^. Experimental studies and a small cross-sectional study recently found that hepatic de novo lipogenesis might be involved in the downregulation of SHBG synthesis and obesity at least in women^30,31^. As the improvement of insulin resistance usually concurs with the decrease in pancreatic insulin production, insulin may also play a role in inhibiting the hepatic production of SHBG.

Leptin is an adipocyte-derived hormone that exerts powerful effects both centrally and peripherally. Multiple leptin-mediated pathways have been described in breast cancer cells, that have been linked to proliferation, cell migration, and increase in therapeutic resistance^32^. Importantly, exemestane had a favorable effect on the adipokine profile and no increase in insulin resistance in our study. Strikingly, in obese women, leptin decreased markedly in all three arms, and no difference was observed between QD and TIW. Thus, although aromatase inhibition has been linked to impaired lipid metabolism and insulin resistance in some studies^33^, exemestane showed a favorable modulation of leptin in obese women. The results seem to suggest that for primary prevention and early-stage breast cancer, we do not need to achieve complete estrogen suppression for therapeutic success. Total aromatase suppression is likely to increase insulin resistance which long term is associated with an increased risk of coronary heart disease as well as promotion of breast cancer development and endocrine resistance. Particularly with the obesity epidemic in the United States, postmenopausal women are likely to die of coronary disease^34^ rather than breast cancer.

However, the mechanism behind a decrease in leptin levels is complex. Androgens have been suggested to decrease plasma leptin levels, while estrogens are believed to increase leptin levels through transcriptional activation of the *LEP* gene^35^. Thus, the estrogen fall associated with aromatase inhibitors likely contributes to the decrease. Estrogen regulates adipose tissue distribution, and the estrogen fall has been associated with an increased risk of visceral fat accumulation^36^, through complex steroid and adipokine signaling^36–38^. The correlation between estrogen and leptin levels is mainly linked to leptin synthesis in the subcutaneous adipose tissue. Two studies have found distinct effects of exemestane, as compared to non-steroidal aromatase inhibitors, on circulating adipokines^39,40^, potentially due to the weak androgen effects of exemestane and its major metabolite 17-hydro-exemestane. Additionally, the metabolite 17-hydro-exemestane has been shown to bind to the androgen receptor (AR) with high affinity^41^. The AR is present in human preadipocytes and adipocytes suggesting that the exemestane metabolite might contribute to down-regulate the transcription of leptin in the adipose tissue^42^. The evidence from our study further underlines an interaction between obesity and biomarker response to exemestane. The expected correlation between estradiol and PgR change on Ki-67 change was observed only in non-obese, and not in obese women. This lack of correlation in obese women points to additional mechanisms interfering with the antiestrogenic activity of exemestane, including insulin resistance and inflammation. Aromatase expression in the breast tissue has been described to be significantly higher in postmenopausal women compared with premenopausal women and associated with BMI and white adipose tissue inflammation^43^. In consideration of the exploratory nature of this study, due to the relatively low number of obese subjects as opposed to non-obese, larger cohorts are needed to confirm the potentially favorable results of intermittent dose exemestane in obese women at risk of breast cancer.

In conclusion, exemestane 25 mg TIW demonstrated similar reductions in the risk biomarkers estradiol, and leptin as the standard daily regimen in obese women. Although reductions in Ki-67 and estrone were more marked with the daily dose, a significant change was also observed for TIW dosing. Taken together our findings support further study of TIW exemestane for breast cancer prevention in obese as well normal weight women.

## Methods

### Study design

Details on this international multicenter, presurgical, 3-arm, double-blind, noninferiority phase IIb trial, including inclusion and exclusion criteria, and power analysis calculation have been published elsewhere^44^. Briefly, postmenopausal women, with histologically-confirmed ER-positive (≥10%) primary breast cancer (cT0 – 2, cN0 – 1) irrespective of Ki-67, were randomized (1:1:1) to either exemestane 25 mg QD (*n* = 57), 25 mg TIW (*n* = 57) or 25 mg QW (*n* = 62) for 4–6 weeks before surgery, with stratification by center and BMI < 25 kg/m^2^ vs ≥25 kg/m^2^ to prevent possible imbalances in estradiol levels among arms. Based on the primary endpoint, a total sample size of 162 participants had 80% power to detect a non-inferiority of −6% in mean percentage change of serum estradiol in the lower dose regimens compared to the standard dose. Assuming a 10% dropout rate, 180 participants had to be randomized. Study completion was high. Overall, four subjects dropped out, due to personal reasons (*n* = 2), one adverse event, and one severe adverse event unrelated to study treatment. The protocol and its amendments have been approved by the National Cancer Institute (NCI) Central Institutional Review Board (IRB) and the local Italian IRBs (registered at Clinical Trials.gov NCT02598557 and IEO 370 EudraCT 2015 – 005063 – 16). All participants signed a written informed consent document. The study was conducted in compliance with all relevant ethical regulations including the Declaration of Helsinki.

### Biomarkers assessment

Morning fasting serum samples were frozen at baseline and final visit (day before surgery) and stored in aliquots at −80 °C until assayed, and freeze and thawed cycles were avoided. Serum adiponectin and leptin were measured using an automated immunoassay platform called ELLA (ProteinSimple, Bio-techne, Minneapolis, MN, USA). The platform is based on a microfluidic technology that allows the performance of automated enzyme-linked immunoassays without manual steps^45^. The inter-assay coefficient of variation of our in-house prepared pooled serum sample was 6.6% for adiponectin and 5.4% for leptin. Sex hormone binding globulin (SHBG) serum levels were measured by a chemiluminescent immunoassay designed for the IDS-iSYS Multi-Discipline Automated System (Immunodiagnostic Systems Limited, United Kingdom), having a lower limit of detection (LLOQ) of 0.30 nmol/L. Insulin was measured by a chemiluminescent microparticle immunoassay technology designed for the ARCHITECT automated instrument (Abbott Diagnostics), having an analytical sensitivity below 1.0 μU/mL. The inter-assay coefficient of variation of ARCHITECT controls (3 different levels) for insulin was below 1.7% and our in-house pooled serum control was 3.7% (mean concentration: 5.2 μU/mL). At baseline and final visits, glucose concentrations were determined locally at each participating center. We applied the homeostasis model assessment (HOMA) as a surrogate index of insulin sensitivity, obtained by the formula [fasting insulinemia (mU/L) × glycemia (mmol/L)]/22.5.

Serum concentrations of estradiol, estrone, exemestane, and 17-hydroxy-exemestane were determined simultaneously using a highly sensitive, previously described assay^46^. In short, samples were spiked with isotope-labeled internal standard and extracted with hexane: methyl tert-butyl ether and hexane: isopropyl alcohol in an automated procedure using a Hamilton Star robot. Extracts were reconstituted and analyzed by LC-MS/MS. The LLOQs were 0.8 pmol/L (estradiol), 0.2 pmol/L (estrone), 13 pmol/L (exemestane), and 8 pmol/L (17-hydroxy-exemestane). Arbitrary values of 0.4 pmol/L for estradiol (*n* = 70), 0.15 pmol/L for estrone (*n* = 7), 6.5 pmol/L for exemestane (*n* = 16); 4 pmol/L for 17-OH-exemestane (*n* = 38); were assigned to levels below LLOQ. The formula used was LLOQ/2.

Testosterone and androstenedione were analyzed using a previously published method^47^. Shortly described, serum samples were spiked with isotope-labeled IS and extracted with ethyl acetate: hexane (80:20). The supernatant was transferred to a new vial and washed with ammonium formate buffer. The organic phase was transferred to a new vial and evaporated under nitrogen gas. Samples were reconstituted and analyzed using LC-MS/MS. The LC-MS/MS system was comprised of a 1290 UPLC system (Agilent) and an API 5500 (SCIEX). The column used was a Zorbax C18. The mobile phases were water and acetonitrile with 0.1% formic acid. The LLOQ was 0.1 nmol/L for testosterone and 0.2 nmol/L for androstenedione.

### Immunohistochemistry

Pre- and post-treatment measurements were centralized at IEO to minimize the variability among different centers. Specifically, immunostainings were performed on formalin-fixed and paraffin-embedded sections of invasive tumors obtained from biopsies and surgical specimens, using anti-ER, PgR, (PharmDX), and Ki-67 (MIB1) antibodies, as previously reported by our group^48,49^.

### Statistical methods

We presented median values and interquartile ranges of serum exemestane, 17-OH-exemestane, circulating estrogens, androgen metabolites, adipokines, insulin and HOMA index and tumor Ki-67 index at baseline and changes from baseline to post-treatment, by arms and BMI. Patients were categorized as obese (BMI ≥ 30 kg/m^2^) and non-obese (BMI < 30 kg/m^2^). The decision to use 30 instead of 25 as a cut-off was based on the proportion of women with obesity higher than expected. From a clinical point of view, the implications of obesity appear more relevant than those of overweight. A supplementary table 3 illustrates the median and interquartile ranges of drug, estradiol, and Ki-67 according to all three BMI categories. Differences at baseline were evaluated by Wilcoxon rank tests. Differences by arms of changes in time were evaluated through ANCOVA models adjusted for baseline values, BMI, and age. The normal distribution of residuals from the full model was graphically checked. Predicted and observed Ki-67 absolute change values were plotted against the % change of serum estradiol by arms and BMI. We also compared the proportions of participants having an absolute decrease in tumor Ki-67 index of at least 3% at the surgery by treatment arms with Chi-square tests. This 3% threshold in Ki-67 decrease after short-term neoadjuvant tamoxifen was shown to be predictive of recurrence-free survival and overall survival^16^. Adjustment for multiple testing was not carried out given the exploratory nature of the subgroup analyses by BMI

### Supplementary information

Supplementary Tables

Related Manuscript File

## Data Availability

Deidentified participant data underlying this article will be shared on reasonable request to the NCI at the following website: https://cdas.cancer.gov/learn/eppt/browse/. A specific purpose for data request is required. The request will be evaluated by the NCI. The availability of any leftover materials (serum, whole EDTA blood, and frozen breast tissue) has to be checked. The material may be shared on reasonable request to the NCI at the following website: https://cdas.cancer.gov/learn/eppt/browse/. The request will be evaluated by the NCI and an Ethical Committee.
