# Supplementary material for: Alternative dosing regimen of exemestane in a randomized presurgical trial: the role of obesity in biomarker modulation
Source: NPJ Breast Cancer. 2024 Jan 18;10:7. doi: 10.1038/s41523-024-00616-8 (PMC10796398; doi:10.1038/s41523-024-00616-8)
Supplement: Supplementary file 1 — Supplementary Tables [file 41523_2024_616_MOESM1_ESM.pdf]

Supplementary Table 1. Median and interquartile ranges of androgen metabolites at baseline and absolute changes by BMI

	**Exemestane 25 mg QD**		**Exemestane 25 mg TIW**		**Exemestane 25 mg QW**		**P-value BMI<30**		**P-value BMI≥30**	
Variable	BMI<30 (n=34)	BMI≥30 (n=19)	BMI<30 (n=38)	BMI≥30 (n=16)	BMI<30 (n=37)	BMI≥30 (n=22)	QD vs TIW	QD vs QW	QD vs TIW	QD vs QW
Testosterone baseline (ng/mL)	0.350 (0.193; 0.659)	0.540 (0.378; 0.877)	0.489 (0.326; 0.804)	0.522 (0.336; 0.874)	0.503 (0.316; 0.752)	0.324 (0.225; 0.603)	1	0.162	0.905	**0.027**
Testosterone absolute change	0.028 (-0.091; 0.112)	0.052 (-0.024; 0.284)	0.001 (-0.256; 0.120)	0.026 (-0.132; 0.060)	0 (-0.107; 0.168)	0.049 (-0.083; 0.257)	0.821	0.116	0.942	0.982
Androstenedione baseline (ng/mL)	1.5 (1.0; 2.0)	1.4 (1.1; 1.7)	1.8 (1.1; 2.7)	1.4 (1.1; 1.8)	1.6 (1.4;2.6)	1.2 (1.1; 1.3)	0.192	0.326	0.621	0.156
Androstenedione absolute change	-0.1 (-0.2; 0.3)	0 (-0.3; 0.3)	0.2 (-0.2; 0.6)	0.2 (-0.2; 0.5)	0.3 (-0.3; 0.8)	0 (-0.1; 0.4)	0.262	0.271	**0.035**	0.819

Supplementary Table 2. Representativeness of Study Participants

Cancer type	Histologically-confirmed ER-positive (≥ 10%) primary breast cancer (cT0–2, cN0–1, Mx)
**Considerations related to:**	
Sex	The incidence of male endocrine-responsive breast cancer is very low, and their inclusion into this three-arm study design was not feasible.
Age	The study included postmenopausal women only. The median age at the time of study entry was 66 years in the QD arm, 63 years in the TIW arm, and 65 years in the QW arm, which is very close to all breast cancer diagnoses, which is around 63
Race/ethnicity	Our study is a phase IIb biomarker trial. As such it is not powered to assess the impact of racial/ethnic or ancestry-based differences in efficacy and toxicity.<br>All five centers were encouraged to include minority races and ethnicities in the study. It should be considered that in Italy, only two hospitals recruited about half of the cohort, all being white, except for 1 black or African.<br>The USA cohort included 1 American Indian, 6 Asians, 1 native Hawaiian, 7 black or African, and 5 of unknown race.<br>Overall, 155 were white (88% of the whole cohort). Thus, some races/ethnicities may be under-representative.
Countries	The two Italian centers were located in Northern Italy. Anyhow, the IEO is an important Centre of Excellence for the prevention, diagnosis, and treatment of breast cancer, with a large number of recruited breast cancer patients coming from other parts of Italy.<br>In the US, the recruiting centers were located in New York, Florida, and Texas.
Overall representativeness of this study	Our study is a phase IIb biomarker trial conducted in 3 US centers and 2 Italian centers, encouraged to include minority races and ethnicities. Despite this effort, it cannot be considered overall representative of US racial/ethnic. The average age distribution of our study is similar to the average age distribution of breast cancer incidence, excluding younger women.

Supplementary Table 3. Median and interquartile ranges of post-treatment serum exemestane, 17-OH- exemestane, and baseline and absolute changes in estradiol, and breast tumor Ki-67 by three BMI categories (normal weight, overweight, and obese)

	Exemestane 25 mg QD			Exemestane 25 mg TIW			Exemestane 25 mg QW		
**Variable**	**BMI<25 (n=17)**	**BMI 25-29.9 (n=17)**	**BMI≥30 (n=19)**	**BMI<25 (n=17)**	**BMI 25-29.9 (n=21)**	**BMI≥30 (n=16)**	**BMI<25 (n=18)**	**BMI 25-29.9 (n=19)**	**BMI≥30 (n=22)**
Exemestane (pmol/L) post-treatment	2858 (1529; 4384)	3034 (2430; 4770)	3355 (2922; 4567)	473 (322; 696)	584 (434; 975)	541 (357; 682)	26 (20;34)	30 (6; 79)	22 (17; 35)
17-OH-exemestane (pmol/L) post-treatment	990 (678; 1217)	832 (546; 1454)	1419 (600; 1865)	165 (84; 246)	327 (150; 497)	228 (154; 406)	12 (4; 16)	4 (4; 47)	4 (4; 22)
Estradiol baseline (pmol/L)	14 (12;19)	20 (12; 27)	36 (24; 56)	11 (9; 15)	25 (14; 35)	28 (22; 46)	12 (10; 17)	18 (12; 29)	24 (22; 38)
Estradiol absolute change	-12.0 (-17.0; -10.7)	-20.0 (-26.0; -12.0)	-35.4 (-53.6; -22.6)	-10.6 (-13.6; -7.7)	-25.0 (-33.8; -13.7)	-26.3 (-44.7; -21.6)	-7.8 (-9.6; -4.7)	-13.6 (-26.2; -8.5)	-20.1 (-26.2; -14.9)
Ki-67 baseline (%)	15 (8; 18)	11.0 (9; 16)	11.5 (7; 17)	12 (8; 22)	12 (6; 21)	15 (8; 18)	8 (5; 14)	14 (7; 22)	10.5 (7; 19)
Ki-67 absolute change	-9 (-10; 0)	-5 (-9; -3)	-8 (-13; -3)	-6 (-12; -1)	-5 (-8; -2)	-4 (-9; -2)	-3 (-6; -1)	-2 (-9; 0)	-5 (-8; -2)
